# Ketoprofen as an emerging contaminant: occurrence, ecotoxicity and (bio)removal

**DOI:** 10.3389/fmicb.2023.1200108

**Published:** 2023-08-07

**Authors:** Elena Tyumina, Maria Subbotina, Maxim Polygalov, Semyon Tyan, Irina Ivshina

**Affiliations:** ^1^Perm Federal Research Center of the Ural Branch of the Russian Academy of Sciences, Perm, Russia; ^2^Microbiology and Immunology Department, Perm State University, Perm, Russia

**Keywords:** pharmaceutical pollution, nonsteroidal anti-inflammatory drugs (NSAIDs), ketoprofen, bioremediation, environmental effects

## Abstract

Ketoprofen, a bicyclic non-steroidal anti-inflammatory drug commonly used in human and veterinary medicine, has recently been cited as an environmental contaminant that raises concerns for ecological well-being. It poses a growing threat due to its racemic mixture, enantiomers, and transformation products, which have ecotoxicological effects on various organisms, including invertebrates, vertebrates, plants, and microorganisms. Furthermore, ketoprofen is bioaccumulated and biomagnified throughout the food chain, threatening the ecosystem function. Surprisingly, despite these concerns, ketoprofen is not currently considered a priority substance. While targeted eco-pharmacovigilance for ketoprofen has been proposed, data on ketoprofen as a pharmaceutical contaminant are limited and incomplete. This review aims to provide a comprehensive summary of the most recent findings (from 2017 to March 2023) regarding the global distribution of ketoprofen in the environment, its ecotoxicity towards aquatic animals and plants, and available removal methods. Special emphasis is placed on understanding how ketoprofen affects microorganisms that play a pivotal role in Earth’s ecosystems. The review broadly covers various approaches to ketoprofen biodegradation, including whole-cell fungal and bacterial systems as well as enzyme biocatalysts. Additionally, it explores the potential of adsorption by algae and phytoremediation for removing ketoprofen. This review will be of interest to a wide range of readers, including ecologists, microbiologists, policymakers, and those concerned about pharmaceutical pollution.

## 1. Introduction

Pharmaceuticals were initially developed to promote and maintain human and animal health. However, they have increasingly become a mixed blessing, posing significant challenges to achieving sustainable development goals and presenting a real threat to both humanity and the environment globally (Boxall, 2004; Arnold et al., 2014; Miettinen and Khan, 2022; Wilkinson et al., 2022). In the late 1990s, pharmaceuticals were recognized as contaminants of emerging concern. This group encompasses surfactants, pesticides, (nano)metals, detergents, plasticizers, solvents, industrial and household chemicals as well as detergent surfactants. Their ecological fate in ecosystems is not thoroughly understood or regulated (Picó et al., 2012).

Although pharmaceuticals are found in trace amounts (ng/L to μg/L) in aquatic and terrestrial environments, they have the potential to negatively impact flora and fauna (Gavrilescu et al., 2015; Wilkinson et al., 2016; Sonne et al., 2021; Hejna et al., 2022; Khan et al., 2022). Furthermore, the continuous discharge of pharmaceuticals can lead to high cumulative concentrations, and the simultaneous coexistence of multiple contaminants in the environment can amplify their toxic effects, creating a more dangerous mixture compared to individual pollutants present alone (Godoy and Kummrow, 2017; Kafaei et al., 2018; Grabarczyk et al., 2020; Ramírez-Morales et al., 2020).

Pharmaceutical pollutants are antidepressants, hormones, antimicrobial and antidiabetic agents, analgesics, and nonsteroidal anti-inflammatory drugs (NSAIDs) (Aus der Beek et al., 2016; Ebele et al., 2017; Khan et al., 2020; Wilkinson et al., 2022). NSAIDs, a class of non-opioid drugs with analgesic, antipyretic, and anti-inflammatory properties, are commonly used to address both acute and chronic pains associated with inflammatory conditions (Panchal and Prince Sabina, 2023). Recently, NSAIDs such as diclofenac, ibuprofen, and naproxen have gained attention due to their widespread presence in the environment, potential ecotoxicity, and the development of removal techniques (Mejía-García et al., 2020; Tyumina et al., 2020; Wojcieszyńska and Guzik, 2020; Jan-Roblero and Cruz-Maya, 2023). Ketoprofen (KTP), another common NSAID found in ecosystems, has also been identified as a potential concern, requiring targeted eco-pharmacovigilance (Wang et al., 2018). KTP (CAS # 22071-15-4; C_16_H_14_O_3_; 2-(3-benzoylphenyl)propanoic acid) is a bicyclic NSAID with a significant volume of its production, sales, and consumption in human and veterinary medicine due to its pronounced prolonged anti-inflammatory, analgesic, and antipyretic effects.

Wang et al. (2018) conducted an exhaustive review based on 85 publications from 2010 to 2017, confirming the status of KTP as a pharmaceutical pollutant of significant environmental risks, with a hazard quotient greater than 1.0 for certain ecosystems. The authors proposed several measures to reduce the amount of KTP entering the environment, including thorough monitoring of its presence, reducing the use of KTP-containing drugs in veterinary medicine, and source control. Additionally, there is a pressing need to develop a comprehensive framework for targeted eco-pharmacovigilance, which should imply an interdisciplinary approach, including ecotoxicological and biomonitoring studies. By incorporating different types of interdisciplinary approaches, we can deeepen our understanding of the ecological impact of pharmaceuticals and establish robust strategies for monitoring and mitigating their effects on the environment.

Biomonitoring studies focused on microbial communities are of particular importance because microorganisms (bacteria, archaea, protists, fungi, and microalgae) play a vital role in the functioning of the biosphere (Sagova-Mareckova et al., 2021). However, the study of their responses to the presence of emerging contaminants and pharmaceutical pollutants in particular is often overlooked. It is crucial to pay closer attention to taxonomic rearrangements in microbial communities, metabolic changes in microbial cells, pathogenicity spread, antibiotic resistance as well as changes in the function of environmentally significant microorganisms in ecosystems exposed to pharmaceuticals. This will ultimately deepen our knowledge of the ecological fate of pharmaceutical pollutants and shed light on the processes by which they undergo transformations (Gomes et al., 2020; Crampon et al., 2021; McLain et al., 2022; Wang et al., 2022). In this regard, the studies of stress-tolerant microorganisms have attracted attention because they possess the potential to biodegrade numerous xenobiotics and pharmaceutical pollutants and contribute to current water treatment technologies (AlKaabi et al., 2020; Pátek et al., 2021; Shylla et al., 2021; Bravo and Braissant, 2022; Ivshina et al., 2022). Furthermore, research on stress-tolerant microorganisms addresses fundamental issues related to pharmaceutical metabolism and the protective mechanisms activated in microbial cells upon exposure.

In this comprehensive review, we gathered and analyzed the most up-to-date information available (from 2017 to March 2023) regarding the detection of KTP in various environments, its ecotoxicity towards animals, plants, and microorganisms as well as different approaches for KTP decomposition. Throughout the review, special attention was given to the bioremoval of KTP, considering the roles of bacteria, fungi, enzymes, algae, and plants in the degradation and removal processes. By synthesizing the current knowledge on detection, ecotoxicity, and various removal approaches of KTP, this review provides valuable insights into the environmental impact of KTP and promising strategies for its remediation. The information was gathered through searches conducted on Google Scholar, Scopus, and Web of Science databases.

## 2. Occurrence of ketoprofen in the environment

### 2.1. Factors contributing to the entry of ketoprofen into aquatic ecosystems

Over the past century, global use of water resources has increased sixfold due to population growth, economic development, and changing consumption patterns (UNESCO and UN-Water, 2020). Consequently, preserving water resources, referred to as the “blue gold” and a vital component of sustainable development, is widely recognized as essential. However, pharmaceutical pollution poses a significant threat to aquatic ecosystems and hinders progress towards at least 12 of the 17 Sustainable Development Goals (Domingo-Echaburu et al., 2021). To address this issue, the establishment of organizations monitoring pharmaceuticals in nature is urgently needed. Initiatives have emerged in several countries to introduce Environmental Risk Assessment (ERA) for pharmaceuticals (Jose et al., 2020) and to establish targeted eco-pharmacovigilance for selected pharmaceutical compounds including KTP (Wang et al., 2018). Below are the factors that determine the entry of KTP into the environment, primarily into aquatic ecosystems.

KTP is ubiquitously used in both human and veterinary medicine for treating pain, inflammation, and fever. KTP is weakly selective for cyclooxygenase isoenzymes (COX-1). The role of COX is to catalyze the formation of prostaglandins from arachidonic acid, which is released from membrane phospholipids upon activation of phospholipase A2 under various stimuli such as inflammatory, physical, chemical, and mitogenic conditions (Tomić et al., 2017). Consequently, KTP is prescribed for treating musculoskeletal disorders, including osteoarthritis and rheumatoid arthritis, and other conditions that cause pain and inflammation. It is also used to relieve mild to moderate pain, such as headaches, dental pain, menstrual cramps, and postoperative pain. In veterinary practice, KTP is used to reduce fever and inflammation in cattle (De Koster et al., 2022), postoperative rehabilitation of dogs (Monteiro et al., 2019; Ravuri et al., 2022), and even to treat injured sea turtles (Harms et al., 2021). Research is ongoing to study the efficacy of KTP for treating other animal species, leading to a wider application range of this drug (Hu et al., 2021; Reynolds et al., 2021). In addition, the drug is being studied for its use as a safe alternative to opioid analgesics (Doleman et al., 2021; Herzig et al., 2021; Pergolizzi et al., 2021). Thus, the volumes of production and consumption of KTP will only increase in the future. This is also facilitated by some stagnation of the pharmaceutical industry and drug shortages (Sharma et al., 2022). Thus, maintaining population health and extending healthy life expectancy is achieved by increasing the production and distribution of existing medications to the population (Mezzelani et al., 2018; Iijima et al., 2021; Locherty et al., 2021; Firdous et al., 2022).

Another factor to consider is the low rate of KTP decomposition and the inadequate efficiency of sewage treatment plants (Jureczko and Przystaś, 2018), resulting in the accumulation of KTP primarily in aquatic ecosystems (Chavoshani et al., 2020; Parolini, 2020; Mejías et al., 2021; Rastogi et al., 2021). KTP tends to accumulate in wastewater, especially hospital wastewater, due to its excretion from humans and animals in the form of metabolites, with only 1% of KTP being excreted unchanged (Kotwani et al., 2021). Moreover, there is a lack of sufficient methods for the proper removal, storage, and disposal of expired or unused KTP. Frequently, pharmaceuticals are flushed down the drain or disposed of in solid waste landfills because they are expired or no longer needed (Kleywegt et al., 2019; Petrie and Camacho-Muñoz, 2021; Rogowska and Zimmermann, 2022). Once KTP enters wastewater treatment plants (WWTPs), it can migrate into surface and groundwater because of poorly developed micropollutant removal technologies (Almeida et al., 2020; Rastogi et al., 2021).

In today’s world, the COVID-19 pandemic has also become a factor promoting a dramatic increase in NSAID concentrations in the environment. The efficacy of NSAIDs in alleviating pain and mitigating inflammatory symptoms associated with coronavirus disease 2019 has been a subject of considerable scientific investigation. Moreover, thorough assessments were conducted to assess the risks associated with the utilization of NSAIDs for this particular condition (Cumhur Cure et al., 2020; Robb et al., 2020; Kelleni, 2021). Certain NSAIDs proved to be effective in relieving COVID-19 symptoms without causing harm, but the long-term effects of taking anti-inflammatory drugs in conjunction with a viral infection remain unclear (Zhou et al., 2022). As a result of the global use of medications during the pandemic since the end of 2019, the detection of NSAIDs in aquatic systems has increased worldwide, with their concentrations ranging from a few nanograms to hundreds of micrograms per liter (Wojcieszyńska et al., 2022).

### 2.2. Detection of ketoprofen in different regions

KTP has been detected in surface water (rivers, lakes, seas, oceans), wastewater (municipal, hospital, industrial), groundwater, and drinking water sources in over 30 countries worldwide (Table 1). Its global occurrence is evident, since trace amounts of KTP have been found in seawater in Antarctica (Szopińska et al., 2022). KTP concentrations are measured in the range of 0.16 ng/L (Italy, Turin, groundwater) to 260 μg/L (India, influent wastewater), with the highest concentration observed in India, where KTP is available over-the-counter. India, a major hub for drug manufacturing, also contributes to pharmaceutical waste discharge into aquatic ecosystems, with only 31% of wastewater undergoing pre-treatment (Philip et al., 2018; Praveenkumarreddy et al., 2021). In Africa, screening of KTP in water has only been conducted in South Africa (Supplementary Figure S1), leaving the extent of KTP contamination across most of the continent unknown (Waleng and Nomngongo, 2022). Similarly, limited research has been carried out in Australia regarding the detection of KTP: only a few sporadic investigations have been conducted so far (Świacka et al., 2022). The scarce data about these continents may be attributed to their predominantly arid landscapes, which receive less attention in terms of KTP detection studies. The focus of drug detection has primarily been on aquatic ecosystems, where the occurrence of KTP has been extensively investigated.

**Table 1:** Measured environmental concentrations (MECs) of ketoprofen in various aquatic ecosystems.

Region	Sample location	MEC, ng/L	Reference
**Algeria**: El-Harrach valley	Surface water	<MDL	Kermia et al. (2016)
WWTP (Beni Messous)	Influent wastewater	565.2	
	Effluent wastewater ^MB^	1,034.5	
WWTP (Reghaia)	Influent wastewater	<MDL	
	Effluent wastewater ^MB^	<MDL	
USTHB University	Drinking water	273	
**Antarctica**: Admiralty Bay	Seawater	10–16.6	Szopińska et al. (2022)
**Australia**: Bega Valley	Influent wastewater	24–15,300	Petrie and Camacho-Muñoz (2021)
	Effluent wastewater ^MBR^	1–6	
	Effluent wastewater ^AS^	24–177	
**Brazil**: Iguaçu River	Surface water	17–620	Chaves et al. (2021)
Minas Gerais		<73–1,020	
River Manus	Surface water	14,215	Rico et al. (2021)
River Macapa		<10–42	
River Belem		<10–69	
**Canada**: The province of Ontario	Influent wastewater (after funeral homes)	56–900	Kleywegt et al. (2019)
The Hudson Plains ecozone and four in the Prairies ecozone in Alberta and Saskatchewan	Effluent wastewater	6.2–77.3	Schwartz et al. (2021)
	Drinking water	5.5	
**China**: Northern Taiwan Strait	Seawater	3.68–6.7	Fang et al. (2019)
Yangtze River	Surface water	12.13–45.01	Jiang et al. (2019)
	Effluent wastewater	7.32–16.80	
Guangzhou	Groundwater	3.08	Dong et al. (2018)
Dongguan	Groundwater	2.08	
Foshan	Groundwater	0.03	
Dongting Lake	Surface water	<MDL–11.3	Wang et al. (2019)
**Costa Rica**: San Jose Province	Influent wastewater	12,900	Ramírez-Morales et al. (2021a)
	Effluent wastewater	14,700	
Different Regions	Influent wastewater	~600 (median)	Ramírez-Morales et al. (2020)
	Effluent wastewater ^AS^	~400 (median)	
Rivers (the Great Metropolitan Area)	Surface water	~200 (median)	Ramírez-Morales et al. (2021b)
**Croatia**: Sava River	Surface water	0.897–52.7	Česen et al. (2018)
WWTP (Zaprešić, Zagreb and Velika Gorica)	Effluent wastewater ^AS^	53.8–2,460	
**Czech Republic**: River Elbe	Surface water	929.8	Marsik et al. (2017)
Water reservoir Svihov	Influent wastewater	<10–6,500	Vymazal et al. (2017)
	Effluent wastewater ^CW^	<10–1,000	
WWTP (Prague)	Effluent wastewater	180–260	Diaz-Sosa et al. (2020)
**Denmark**: Wetland (Aarslev) and lake (Brabrand)	Surface water	31–62	Kaur et al. (2020)
	Effluent wastewate ^CW^	<6	
**Finland**: WWTP (Taskila)	Influent wastewater	220–730	Leiviskä and Risteelä (2022)
	Effluent wastewater ^MBR^	110–550	
	Effluent wastewater ^CAS^	120–520	
Kenkäveronniemi WWTP (Mikkeli)	Influent wastewater	130–220	Gurung et al. (2019)
**France**: WWTP (Scientrier)	Hospital wastewater	3,600–20,000	Wiest et al. (2018)
	Effluent wastewater ^CAS^	630–900	
WWTP (Seine)	Influent wastewater	1,169 ± 121	Guillossou et al. (2019)
	Effluent wastewater ^AC^	240 ± 47	
**Greece**: WWTP (Athens)	Influent wastewater	822 ± 336	Koumaki et al. (2021)
	Effluent wastewater ^HRAS^	353 ± 61	
	Effluent wastewater ^HRAS^	396 ± 34	
WWTP (Thessaloniki)	Effluent wastewater	7	Ofrydopoulou et al. (2022)
WWTP (Aineias)	Effluent wastewater	23	
**India**: River Ganges	Surface water	107	Sharma et al. (2019)
	Groundwater	<MDL–23.4	
WWTP (Manipal)	Influent wastewater	50–260,000	Praveenkumarreddy et al. (2021)
	Effluent wastewater ^AS^	80–1,350	
WWTP (Udupi)	Influent wastewater	40–37,000	
	Effluent wastewater ^AS^	<MDL–2,710	
WWTP (Mangalore)	Influent wastewater	10–10,500	
	Effluent wastewater ^UASBR^	<MDL–2,700	
Gurupura River	Surface water	10–9,090	
River Ganges	Surface water	<MDL–245	Singh and Suthar (2021)
Yamuna River	Surface water	8,070 (max)	Mishra et al. (2023)
**Italy**: River Lambro	Surface water	0.9–30.8	Castiglioni et al. (2018)
WWTPs (Milan)	Influent wastewater	708–1,924; 792–1,242; 685–1,283	
	Effluent wastewater	25–220; 13–735; 61–316	
Turin	Groundwater	0.16–152.98	Papagiannaki et al. (2021)
	Surface water	0.43–71.84	
Different regions	Influent wastewater	<50–5,150	Di Marcantonio et al. (2023)
	Effluent wastewater	<50–2,420	
Ligurian Coast	Seawater	147–308 (mean)	Benedetti et al. (2022)
**Japan**: WWTP (Sapporo)	Effluent wastewater ^AS^	1,170	Hara-Yamamura et al. (2022)
	Effluent wastewater ^MBR^	490	
Rivers (different regions)	Surface water	150.1	Suzuki et al. (2021)
**Malaysia:** Selangor state	Influent wastewater	29,400	Mohd Hanafiah et al. (2022)
Selangor Darul Ehsan	Influent wastewater	23,900	Mohd Hanafiah et al. (2023)
	Effluent wastewater	9,900	
Langat River	Surface water	10.6	
	Treated water	4.4	
**Mexico**: Hospital in San Nicolas de los Garza	Hospital wastewater	320–980	Hernández-Tenorio et al. (2021)
Amacuzac River	Surface water	2.5–8.3	Rivera-Jaimes et al. (2018)
WWTP (Acapantzingo)	Influent wastewater	23–78	
	Effluent wastewater ^CAS^	9.2–30	
**Morocco:** River Bouregreg	Surface water	30–198	Chafi et al. (2022)
**Pakistan**: Drainage system in Lahore (Shama Drain and Cantonment Drain)	Effluent wastewater	81–132	Ashfaq et al. (2019)
**Poland**: Drwina River	Surface water	5–99	Styszko et al. (2021)
WWTP (Plaszow)	Influent wastewater	8,100 ± 800	
	Effluent wastewater ^AS^	<MDL	
WWTP (Kujawy)	Influent wastewater	10,700 ± 7,300	
	Effluent wastewater ^AS^	<MDL	
Wierzyca River	Surface water	<MDL–25	Stepnowski et al. (2020)
Warta River	Surface water	<MDL–47	
**Portugal**: Ave River	Surface water	50–217	Barbosa et al. (2018)
Cértima River	Surface water	702	
WWTP (Coimbrão)	Influent wastewater	0.5–1.6	Paíga et al. (2019)
	Effluent wastewater ^AS^	0.26–0.88	
Amieira River	Surface water	27.47–112.64	Palma et al. (2020)
Zebro River	Surface water	68.92–321.40	
Álamos River	Surface water	14.72–38.49	
Lucefécit River	Surface water	12.88–43.91	
**Russia**: The Gulf of Finland	Seawater	1.5–4,451.6	Chernova et al. (2021)
WWTPs (St. Petersburg)	Effluent wastewater	756	
	Influent wastewater	267	
**Slovenia**: WWTP	Influent wastewater	0.534–692	Česen et al. (2018)
	Effluent wastewater ^AS^	0.536–24.3	
**South Africa**: Msunduzi River	Surface water	<MDL–437	Agunbiade and Moodley (2016)
WWTP (Amanzimtoti)	Influent wastewater	28,400	Zunngu et al. (2017)
	Effluent wastewater	3,500	
WWTP (Kingsburgh)	Influent wastewater	28,200	
	Effluent wastewater	3,400	
WWTP (Umbilo)	Influent wastewater	27,300	
	Effluent wastewater	2,900	
WWTP (Daspoort)	Influent wastewater	<MDL–23,100	Mhuka et al. (2020)
	Effluent wastewater	<MDL–49,480	
Apies River	Upstream frequency	<MDL–8,853	
	Downstream frequency	<MDL–39,490	
WWTP (Johannesburg)	Influent wastewater	159,000	Akawa et al. (2020)
	Effluent wastewater	91,100	
River (Johannesburg)	Surface water	23,800	
Umgeni River	Surface water	0–9,220	Gumbi et al. (2017)
**Spain**: Turia River	Surface water	22–686	Ccanccapa-Cartagena et al. (2019)
Tagus River	Surface water	<1.5–2,149	Rico et al. (2019)
The Besòs River Delta	Groundwater Surface water	97.7 (mean) 9.3	Jurado et al. (2021)
Ebro River	Surface water	39.2	Čelić et al. (2019)
WWTPs (Amposta^AS^ and Sant Carles de la Ràpita ^CAS^)	Effluent wastewater ^AS/CAS^	<1,500	
Gulf of Cadiz	Seawater	<MDL	Biel-Maeso et al. (2018)
Cadiz Bay	Seawater	<MDL–2.6	
	Influent wastewater	883–1,118	
	Effluent wastewater	465–505	
Province of Córdoba (Breña dam)	Influent wastewater	88–510	Petrie and Camacho-Muñoz (2021)
	Effluent wastewater ^CAS^	24–177	
Unuversity BarcelonaTech	Influent wastewater	278–2,881	Ávila et al. (2021)
	Effluent wastewater ^CW^	33–1,129	
WWTP (Galindo)	Effluent wastewater	53–281	Mijangos et al. (2018)
WWTP (Gorliz)	Effluent wastewater	5–13	
WWTP (Gernika)	Effluent wastewater	19–374	
WWTPs “Los Vados” (Granada)	Sewage sludge	10.0^*^	Ledezma-Villanueva et al. (2022)
**Sweden**: WWTP (Stengården)	Influent wastewater	155	Ekengren et al. (2020)
	Effluent wastewater ^AC^	223	
Baltic current	Seawater	0.8–2.7	Gustavsson et al. (2017)
**Tunisia**: The Tahar Sfar Hospital	Hospital effluent	200–18,100	Afsa et al. (2020)
WWTPs (Mahdia)	Influent wastewater	1,200–3,300	
	Effluent wastewater ^AS^	350–790	
Coast of the Mediterranean Sea	Seawater	<MDL–76	
Nabeul region	Effluent wastewater Storage basin	417 179	Mahjoub et al. (2022)
**Turkey**: Konya	Hospital effluent	35.3–9,193	Aydin et al. (2019)
	Influent wastewater	38.3–460	
	Effluent wastewater	7.4–122	
**United Kingdom**: Southwest part	Influent wastewater ^TF^	32–39	Petrie and Camacho-Muñoz (2021)
	Influent wastewater ^SBR^	<13	
	Effluent wastewater ^TF^	3–6	
	Effluent wastewater ^SBR^	114–200	
	Enfluent wastewater ^CAS^	12–16	
	Surface water	<0.2–8	

WWTP, wastewater treatment plant; * – ng/g DW (dry weight). Method of wastewater treatment: AC, activated carbon; TF, trickling filter beds; SBR, sequencing batch reactor; MBR, membrane bioreactor; UASBR, upflow anaerobic sludge blanket reactor; CAS, conventional activated sludge; MDL, method detection limits; CW, constructed wetlands; HRAS, high-rate activated sludge; MB, mechanical-biological.

Upon examining the findings from studies on KTP detection, several noteworthy observations were revealed. The southern regions of India and Africa exhibited the highest levels of KTP pollution, with recorded concentrations reaching a maximum of 260,000 ng/L and 159,000 ng/L, respectively (Supplementary Figure S1). In Europe, significant concentrations were detected in specific locations, such as hospital wastewater in France (20,000 ng/L), influent wastewater in Southern Poland (10,700 ng/L), and surface water in the Czech Republic (6,500 ng/L) and Italy (5,150 ng/L). Studies conducted in the Americas are relatively limited, but higher concentrations were found in the effluent wastewater of Costa Rica (14,700 ng/L) and the surface waters of the Manus River in Brazil (14,215 ng/L). In Australia, increased concentrations of KTP were observed in the influent wastewater of Bega Valley, reaching 15,300 ng/L (Table 1).

It is important to note that certain WWTPs demonstrated better efficiency in the removal of KTP. As shown in Table 1, WWTPs incorporating activated sludge (AS, CAS, HRAS, and UASBR) exhibited the highest KTP removal rates, while WWTPs utilizing activated carbon showed the least efficient removal. These findings underscore the variability in treatment performance and highlight the importance of selecting appropriate treatment technologies for effective KTP removal in WWTPs.

In some countries, despite their similar geographic location, variations can be observed in the effectiveness of KTP removal from wastewater. These differences may be attributed to specific wastewater compositions, hydraulic retention times, redox conditions, loading on treatment plants, and filtration types employed (Wiest et al., 2018). Several studies reported fluctuations in KTP concentrations depending on the season during which the samples were collected (Barbosa et al., 2018; Česen et al., 2018; Aydin et al., 2019; Leiviskä and Risteelä, 2022). One possible reason for these fluctuations is the recurring exacerbations of rheumatoid arthritis and osteoarthritis that often occur during the winter and rainy seasons, resulting in an increased demand for KTP (Camacho-Muñoz et al., 2019).

## 3. Ecotoxic effects of ketoprofen

KTP and its metabolites are considered emerging contaminants as they have been detected in the environment relatively recently, and there are no established environmental quality standards for them. The lack of monitoring studies makes it difficult to assess the potential threat of KTP to ecosystems (Miller et al., 2018; European Commission, 2019). To develop adequate ERA procedures, *in vivo* studies are needed to determine the toxicity rates, biological effects, and modes of action of KTP and its metabolites (Diaz-Sosa et al., 2020; Yusuf et al., 2021).

KTP is a chiral compound and exists in the form of a racemate made up of both (*S*) and (*R*) enantiomers. The chirality of a substance plays a crucial role in determining its chemical properties, including its toxic effects (Bayatloo et al., 2022). It is important to evaluate the impact of both (*S*) and (*R*) isomers on living organisms in order to understand which form of the compound poses a greater toxic burden on the environment (Ou-Yang et al., 2022).

Pharmaceutical substances, including KTP, have the potential to exert their effects on living organisms even at low concentrations. They can permeate biological membranes, accumulate within organisms, and induce ecotoxicological effects (OECD, 2019; Rogowska et al., 2020; Mulkiewicz et al., 2021). Ecotoxicity refers to the ability of a substance or physical agent to cause adverse effects on both the environment and organisms, including animals, microbes, and plants. Ecotoxicity testing involves evaluating the response and sensitivity of representative populations of specific species, often aquatic organisms such as algae, arthropods, mollusks, and fish, to the pollutant over a defined period of time (Rogowska et al., 2020).

### 3.1. Acute toxicity of ketoprofen

Ecotoxicological studies aim at determining both acute and chronic toxicity of substances. Acute toxicity refers to harmful effects caused by a single or multiple exposures over a short period of time, often leading to a lethal outcome. Acute toxicity is typically expressed as either the median effective dose (ED_50_) or the median lethal dose (LD_50_). The ED_50_ represents the concentration of exposure that produces a measurable effect in 50% of individuals in a population, while the LD_50_ represents the lethal concentration that causes death in 50% of individuals. To evaluate the potential hazard of pharmaceutical pollutants to aquatic ecosystems, the Global Harmonized System of Classification and Labeling of Chemicals (GHS) is commonly used (United Nations, 2011). According to GHS, substances are classified into the following categories:

(I) highly toxic: LD_50_/ED_50_ ≤ 1 mg/L;
(II) toxic: 1 mg/L < LD_50_/ED_50_ ≤ 10 mg/L;
(III) hazardous to aquatic organisms: 10 mg/L < LD50/ED50 ≤ 100 mg/L;
(IV) non-toxic: LD_50_/ED_50_ > 100 mg/L.

Based on the available data on acute toxicity and applying the GHS classification, KTP is classified as a compound that is hazardous to aquatic ecosystems (Table 2).

**Table 2:** The acute toxicity of ketoprofen towards organisms from different taxonomic groups.

Organism	Exposure, h	Indicator, mg/L		Reference	Hazard category
		ED_50_	LD_50_		
Invertebrates					
*Artemia salina*	48		13.24	Diaz-Sosa et al. (2020)	III
	48	8.98–16.58		Minguez et al. (2016)	II-III
*Ceriodaphnia silvestrii*	48	24.84		Caldas et al. (2021)	III
*Daphnia magna*	24		11.02	Bownik et al. (2020)	III
	48	25.87–42.24		Minguez et al. (2016)	III
Fish					
*Danio rerio*	24	11.7		Neale et al. (2019)	III
*Pimephales promelas*	96		2.9	Gao et al. (2022)	II
Green algae					
*Pseudokirchneriella subcapitata*	96	0.24 (racemic) 0.066 (dexketoprofen)		Mennillo et al. (2018)	I
	72	43.07–51.14		Minguez et al. (2016)	III
*Scenedesmus obliquus*	96		2.20	Wang et al. (2020a)	II
*Lemna minor*	96	1.9		Ramírez-Morales et al. (2022)	II

LD_50_ – the average lethal dose; ED_50_ – the average effective dose; I – highly toxic, II – toxic, III – hazardous for aquatic organisms, IV – non-toxic (United Nations, 2011).

### 3.2. Chronic toxicity of ketoprofen

**Figure 1:**
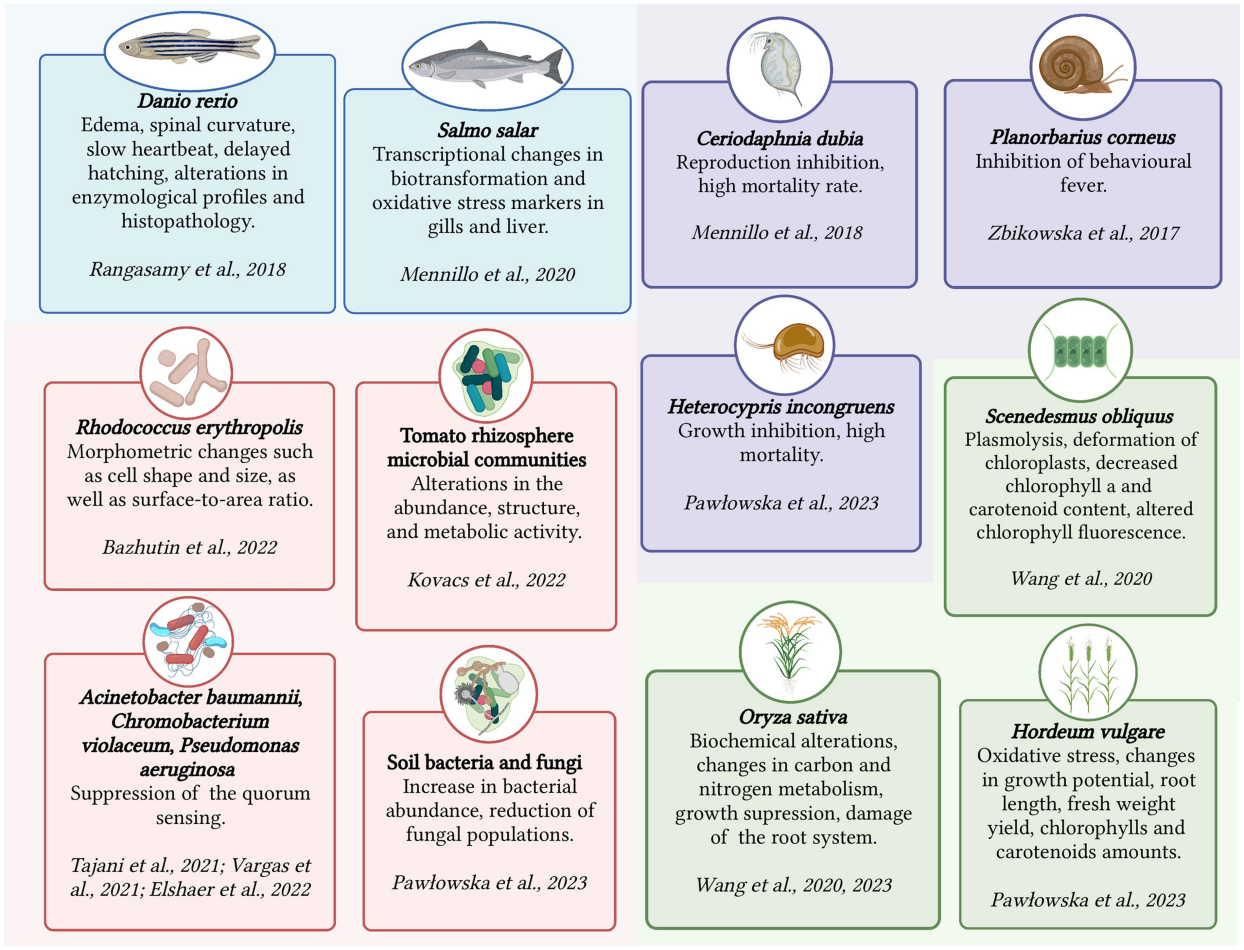
Ecotoxicological effects of ketoprofen on several micro- and macroorganisms. Created with BioRender.com.

Chronic toxicity is an important aspect to consider when assessing the impact of pharmaceutical substances such as KTP on living organisms. Unlike acute toxicity, which focuses on short-term lethal effects, chronic toxicity examines the long-term effects of prolonged exposure to a stressor. Chronic toxicity is characterized by sublethal responses rather than immediate lethality (Parolini, 2020). The assessment of chronic toxicity involves determining the concentration without the observed effect (NOEC) or the lowest observed effect concentration (LOEC). These concentrations serve as criteria for evaluating chronic toxicity and help establish safe exposure levels. Chronic toxicity evaluation encompasses various aspects such as cytotoxicity (cellular damage), genotoxicity (damage to genetic material), disturbances in gene expression and metabolism, endocrine disruption (disruption of hormone systems), teratogenic effects (developmental abnormalities), morphological and tissue transformations, and behavioral changes (Świacka et al., 2021). Figure 1 summarizes the detrimental effects of KTP on several micro- and macroorganisms.

#### 3.2.1. The impact of ketoprofen on vertebrates

The bioaccumulation of KTP in aquatic vertebrates, especially bony fish, is a significant characteristic that influences its behavior in aquatic ecosystems. KTP can be absorbed from the surrounding environment and gradually accumulated in the tissues of these organisms, potentially resulting in higher concentrations as it progresses up the food chain. For instance, KTP has been detected in the heart of Thinlip mullet (*Liza ramada*), a river fish that inhabits environments heavily impacted by WWTP effluents (Manjarrés-López et al., 2023). Furthermore, KTP has been found in the tissues of marine fish, such as Atlantic salmon, Atlantic Sea wolf, and Rainbow trout, at concentrations ranging from 80 to 120 μg/kg (Pashaei et al., 2022a). Another study reported the occurrence of KTP in marine fish, including mullet, white mullet, and snook at concentrations up to 12 ng/kg WW (Mello et al., 2022).

In addition to its bioaccumulation, KTP elicits various negative biological responses in vertebrates. One of the key impacts of KTP is its ability to induce oxidative stress through the formation of reactive oxygen species (ROS). The production of ROS can disrupt the balance of antioxidants and result in oxidative damage to cellular components such as lipids, proteins, and DNA. KTP can also bind to specific enzymes in the body and interfere with their normal catalytic activity, leading to disrupted essential biochemical pathways. Studies on adult *Danio rerio* fish, for example, showed that exposure to KTP led to significantly increased activity of biochemical enzymes and an elevated level of lipid peroxidation in the liver tissues, while the level of antioxidants was significantly reduced (Rangasamy et al., 2018).

The mechanism of action of KTP involves blocking of a cyclooxygenase, which interferes with the normal production of prostaglandins, important regulators of various physiological processes (Rouzer and Marnett, 2020). This disruption can have adverse effects on the intestinal system, where prostaglandins play a role in maintaining gut integrity and function. The interference of KTP with prostaglandin synthesis can promote intestinal toxicity and related gastrointestinal complications. In the case of Atlantic salmon (*Salmo salar*) exposed to KTP (100 μg/L) for 10 days or more, significant impairments in antioxidant responses in the gills and liver damage were observed (Mennillo et al., 2020).

Moreover, KTP has been found to influence nucleotide metabolism, which is crucial for cellular energy production and DNA synthesis (Da Silveira et al., 2013). Disruptions in nucleotide metabolism caused by KTP can impact cellular functions and contribute to the negative biological responses. In *Danio rerio* embryos, increased concentrations of KTP (1, 10, and 100 μg/L) led to developmental changes such as spinal curvature, slowed heart rate, delayed hatching, and even lethal outcomes during a 42-day exposure (Rangasamy et al., 2018).

However, not all studies have shown negative effects of KTP on aquatic fauna. Greene et al. (2020) observed no significant side effects in two species of bony fish (*Oreochromis niloticus* and *Oncorhynchus mykiss*) when administered 3 mg/kg and 8 mg/kg of KTP through intravenous or intramuscular injection. This suggests the potential for KTP applications in aquaculture and pain management in fish. Although the use of analgesics in aquaculture remains limited, improvements in animal welfare have led to elevated interest in pain management in fish (Chatigny et al., 2018). It was previously thought that fish could not feel pain, but modern research has shown that fish, like mammals, have nociceptors that transmit pain stimuli (Sneddon, 2019; Greene et al., 2020).

#### 3.2.2. The impact of ketoprofen on invertebrates

KTP was found to have toxic effects on invertebrates inhabiting water bodies, such as arthropods and mollusks. The exact mechanisms through which KTP exerts its toxicity in invertebrates are not yet fully understood, but this pollutant is believed to interfere with essential cellular processes, including protein production, DNA damage, and generation of ROS. Similar to vertebrates, invertebrates showed bioaccumulation of KTP (Mello et al., 2022; Xu et al., 2022; Pashaei et al., 2022a). Chronic toxicity studies of the racemic mixture of KTP and its *S*-enantiomer using the crustacean *Ceriodaphnia dubia* demonstrated negative effects in a concentration range of 1–1,000 μg/L. These effects included disruptions in the functioning of the reproductive system and a population mortality rate of up to 40% (Mennillo et al., 2018). Treatment with KTP (100 μg/g) led to the suppressed behavioral fever symptoms and reduced motility in the freshwater snail *Planorbarius corneus*. The phenomenon of behavioral fever, which is the rise of body temperature in response to pyrogens, has not been extensively studied in ectothermic animals yet. The results did not provide a clear answer whether the behavioral fever was a similar mechanism to fever in warm-blooded animals. Nonetheless, it could be considered as an endpoint in assessing the effects of xenobiotics on the physiology of invertebrates (Zbikowska et al., 2017).

There is limited data on the migration of pharmaceutical pollutants in living organisms through trophic transfer (Zhang et al., 2021). As for insects with complex life cycles, such as mayflies, they can play a role in the migration of micropollutants from aquatic to terrestrial ecosystems. Mayflies undergo both aquatic and terrestrial stages, allowing them to accumulate and retain micropollutants throughout their life cycle (Grgić et al., 2023). However, research on particular impacts of KTP on terrestrial animals is limited and existing studies are focused on relatively high concentrations. For example, a study examined the impact of KTP and a mixture of KTP and ibuprofen on the growth and mortality of *Heterocypris incongruens*, a common species of crustacean found in sediment and soil (Pawłowska et al., 2023). The results showed that high concentrations (100 mg/kg of soil DW) of these drugs inhibited the growth of the crustaceans, while the drug concentration of 1,000 mg/kg of soil DW caused 100% mortality.

#### 3.2.3. The impact of ketoprofen on plants

KTP has been detected in animal manure as a result of its active use for fever relief in cattle (De Koster et al., 2022). It has also been found in treated wastewater in its original form or as metabolites (see Table 1). This may have negative effects on many plant species as animal manure and purified wastewater are often used as fertilizers and for irrigation in agriculture (Gros et al., 2019; Papaioannou et al., 2020; Ben Mordechay et al., 2021; Kesari et al., 2021; Li et al., 2022). The detrimental effects of KTP on plants can be attributed to its ability to permeate the phospholipid layer of cell membranes and selectively bind to membrane proteins (Mastova et al., 2022). This interaction between KTP and membrane proteins can disrupt their normal functioning. Additionally, under conditions such as photolysis or irradiation, KTP undergoes a process where its ketyl radical is formed. The formation of the ketyl radical is of particular concern as it can initiate lipid oxidation within the cell membrane. This oxidative damage may lead to the disruption of membrane integrity and fluidity, affecting vital cellular processes. Furthermore, the oxidative damage caused by KTP and its ketyl radical can trigger a biochemical cascade within the plant cells, which can have far-reaching consequences. This cascade may involve the generation of reactive oxygen species which might lead to DNA damage, compromising the genetic material and potentially disrupting important cellular functions.

For instance, when exposed to concentrations of 10 and 20 mg/L, KTP had significant effects on rice seedlings (*Oryza sativa* L.) (Wang et al., 2020b). It resulted in changes in photosynthetic pigment content, altered expression of genes related to chlorophyll synthesis and the photosynthetic electron transport chain, hindered growth, and caused damage to the root system. Subsequent metabolomic analysis revealed that KTP negatively impacted the respiration rate, ATP synthesis, and disrupted carbon and nitrogen metabolism, leading to changes in key enzymes involved in these processes (Wang et al., 2023). In another study, KTP and a mixture of KTP and ibuprofen had significant effects on spring barley (*Hordeum vulgare* L.), namely reduced growth potential, root length, fresh weight yield, altered levels of chlorophylls and carotenoids, and induced oxidative stress (Pawłowska et al., 2023).

Some insights were also gained from studies using lesser duckweed (*Lemna minor* L.) at environmentally relevant concentrations of KTP (0.24 μg/L) (Alkimin et al., 2019, 2020). These studies revealed a decrease in the activity of carbonic anhydrase, a crucial enzyme involved in photosynthesis, growth, and development. Conversely, an increase in catalase activity was observed, indicating that plants exhibited adaptive responses to counteract the oxidative stress caused by KTP exposure.

#### 3.2.4. The impact of ketoprofen on microorganisms

KTP, like other NSAIDs, possesses antimicrobial properties and has been investigated for its effects on microorganisms. Previous studies basically focused on determining the acute toxicity of KTP at high concentrations (Marchlewicz et al., 2017; Żur et al., 2018; Petrie and Camacho-Muñoz, 2021; Praveenkumarreddy et al., 2021). For instance, KTP was found to inhibit the quorum sensing of gram-negative pathogens such as *Acinetobacter baumannii*, *Chromobacterium violaceum*, and *Pseudomonas aeruginosa* (Tajani et al., 2021; Vargas et al., 2021; Elshaer et al., 2022). An intriguing work by Neale et al. (2019) aimed to determine the effects of different NSAID enantiomers on the bacterium *Photobacterium leiognathi* using the Bacterial Luminescence Toxicity Screen. However, no statistically significant differences in ecotoxicity measures were found between (*R*)-KTP, (*S*)-KTP, and racemic KTP.

In the case of microalgae, exposure to KTP (1 mg/L) induced various detrimental effects on the green microalga *Scenedesmus obliquus*, including plasmolysis, deformation of chloroplasts, reduced chlorophyll a and carotenoid content, and alterations in chlorophyll fluorescence parameters (Wang et al., 2020a).

Prolonged exposure to KTP can lead to morphological and metabolic changes in microorganisms. For instance, the bacterium *Rhodococcus erythropolis* IEGM 746, which is capable of degrading KTP, exhibited morphometric alterations, such as changes in cell shape, size, and surface-to-area ratio, when exposed to KTP (100 mg/L) for 4 days. These changes represent an adaptive response of the bacteria to counteract the toxic effects of the NSAID (Bazhutin et al., 2022).

In recent years, the scientific community has become increasingly concerned about the potential impact of ongoing exposure to emerging contaminants on natural microbiota (Stadler et al., 2018; Yan et al., 2018; Gomes et al., 2020; Chaturvedi et al., 2021; Pinto et al., 2022; Świacka et al., 2023). Microorganisms respond rapidly to environmental changes, and microbial communities are dynamic organisms that continuously evolve. Changes and rearrangements in soil microbial communities are of particular interest due to their potential detrimental effects: the depletion of rhizospheric microflora, disturbance of ecosystem functioning, and reductions in agricultural crop yields (Cycoń et al., 2016; Raaijmakers and Kiers, 2022; Pashaei et al., 2022b).

Kovacs et al. (2022) have shown that NSAIDs, including diclofenac, ibuprofen, and KTP, can alter the abundance, structure, and metabolic activity of microbial communities in the rhizosphere of tomato plants. The findings suggest that these compounds have the potential to affect the ability of rhizosphere microbiota to provide essential ecosystem services. Another recent study by Pawłowska et al. (2023) investigated the effects of KTP and a mixture of ibuprofen and KTP on bacterial and fungal abundance in soil. The results revealed an increase in bacterial abundance after 14 days of introducing KTP and the mixture into the soil. However, KTP had an adverse effect on the fungal population at a concentration of 1,000 mg/kg.

It’s worth noting that microbial communities consisting of bacteria and fungi are commonly found sharing microhabitats, and their interactions within these environments are dynamic and co-evolving (Deveau et al., 2018). However, the specific features of these bacterial-fungal interactions under the influence of pharmaceutical pollutants are not fully understood. Further research in this area could provide valuable insights and potentially lead to new strategies for the bioremediation of natural ecosystems affected by pharmaceutical pollutants (Khan et al., 2023).

## 4. Bioremoval of ketoprofen

In recent years, increasing emphasis has been placed on the discovery and development of effective methods to neutralize and detoxify pharmaceutical compounds. To address this challenge, it is important to consider various factors that influence the decomposition process of pharmaceutically active compounds. These are physicochemical properties of pharmaceuticals (hydrophobicity and dissociation), environmental conditions (pH, temperature, and aeration), particle size, and others (Nguyen et al., 2021). When it comes to KTP, its complex composition and the presence of various functional groups (methyl, ketone, and carboxylic) contribute to its weak sorption and strong desorption capabilities (Yu et al., 2011). The carboxylic functional group (-COOH), which acts as an electron acceptor, creates an electron deficit, making the compound less susceptible to oxidative catabolism (Dalecka et al., 2020).

Biological neutralization of KTP has garnered significant attention, as it can account for up to 99% of the total removal of KTP during wastewater treatment (Di Marcantonio et al., 2023). Several strategies have been developed and tested for the bioremoval of pharmaceutical compounds from natural environments. The current review compiles the latest knowledge on modern methods and technologies for eliminating KTP from natural environments using fungi, bacteria, algae, and microbial consortia.

### 4.1. Fungal degradation of ketoprofen

Fungi have been shown effective in removing xenobiotics due to the presence of low-specificity enzymes like peroxidases, laccases, and cellulases, which can modify and break down lignin. Ascomycetes and basidiomycetes often use the metabolic pathways of lignin degradation as a tool for the biological degradation of various targets, including pharmaceutical pollutants (Rastogi et al., 2021; Tormo-Budowski et al., 2021).

**Figure 2:**
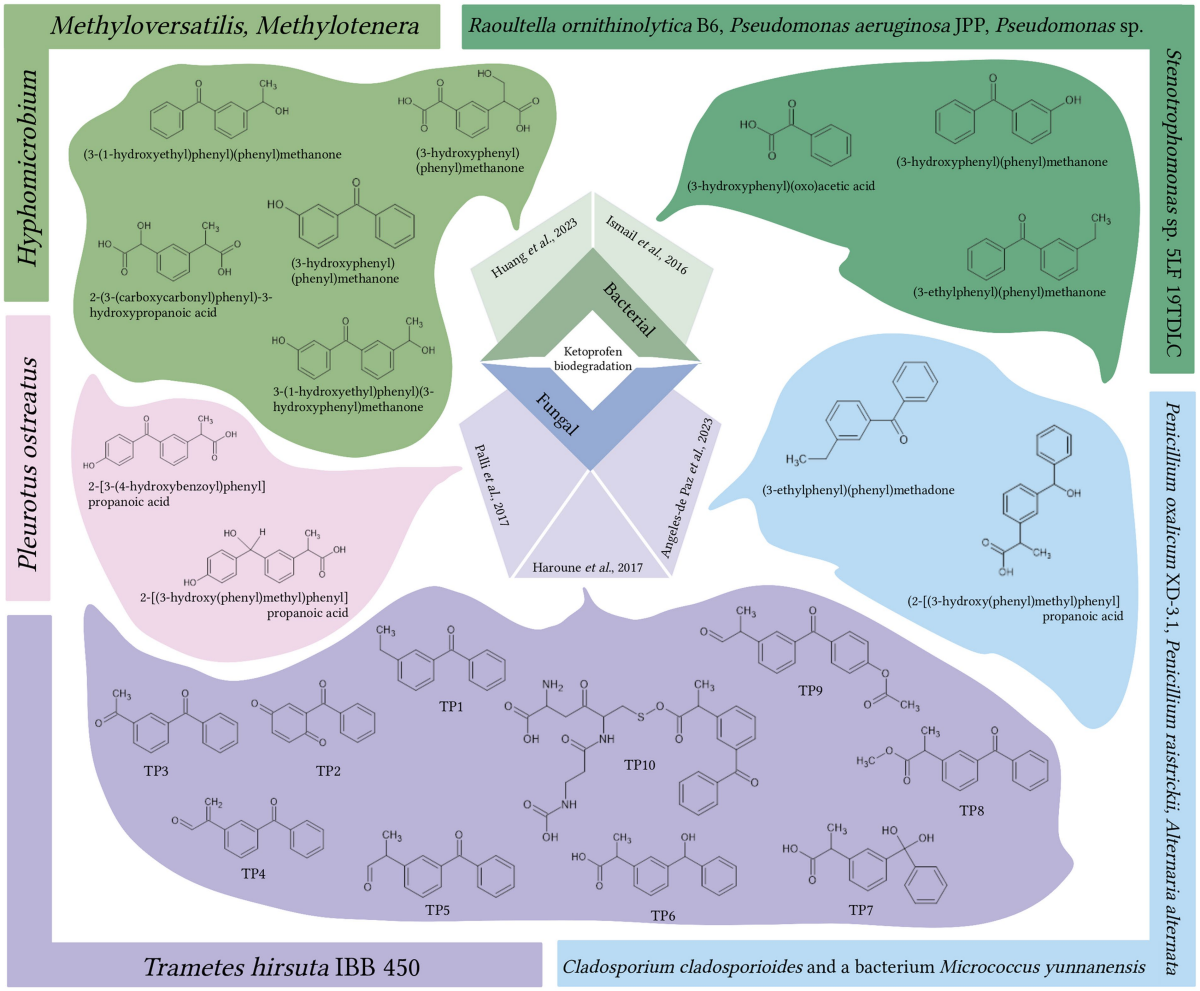
Metabolites generated through the bacterial and fungal biodegradation of ketoprofen (Ismail et al., 2016; Haroune et al., 2017; Angeles-de Paz et al., 2023; Huang et al., 2023), TP – transformation product. Bacterial or fungal biodegraders and their corresponding metabolites depicted in matching colors. Created with BioRender.com.

Among fungi, white-rot fungi have gained significant popularity in bioremediation efforts (Bulai et al., 2018; Sośnicka et al., 2022). Representatives of *Trametes* spp., *Pleurotus* spp., *Phanerochaete* spp., *Irpex* spp. have already proven themselves as promising biodegraders of pharmaceutical pollutants, including NSAIDs (Kum et al., 2009; Marco-Urrea et al., 2009, 2010; Hata et al., 2010; Rodarte-Morales et al., 2011; Nguyen et al., 2013; Sośnicka et al., 2022). In a study by Haroune et al. (2017), *Trametes hirsuta* IBB 450 degraded most (90%) of the 100 μg/L mixture of NSAIDs (acetaminophen, ibuprofen, indomethacin, KTP, mefenamic acid, and naproxen) within a 48-h experiment. The biodegradation of KTP was a multi-step process that involved uptake, intra- and extracellular transformation. The authors also proposed 10 products of KTP transformation (Figure 2), although further structural elucidation methods are required to confirm these proposed structures (Haroune et al., 2017).

Immobilization is often employed as an effective approach to accelerate and optimize the bioremediation of contaminants. Comparative experiments conducted by Tormo-Budowski et al. (2021) demonstrated that the use of immobilized *Trametes versicolor* strain ATCC 42530 on rice husks in fungal bioreactors, such as trickle-bed and stirred tank systems, increased the removal efficiency of 16 pharmaceuticals (1000 μg/L) from hospital wastewater. In a 336-h experiment, the stirred tank bioreactor removed approximately 54% of KTP, while the trickle-bed bioreactor with immobilized fungi removed over 99% of the pharmaceutical pollutant, with 73.3% being due to adsorption. Additionally, toxicological tests revealed a reduction in the toxicity of the treated wastewater after processing in the trickle-bed bioreactor (Tormo-Budowski et al., 2021).

In another study, Torán et al. (2017) found that the strain ATCC 42530, immobilized on wood granules in a fluidized bed bioreactor, was able to remove 80% of KTP (20 mg/L) from synthetic tap water over 31 days. The maximum loss of this NSAID was achieved on day 18 of the experiment in flocculated hospital wastewater. The authors concluded that immobilization on wood was an excellent strategy to maintain the high functional activity and survival of the fungus since pallet wood could be used as a specific source of nutrients. Based on these findings, the authors investigated the possibility of removing KTP in a packed-bed bioreactor under continuous mode of operation, and were able to achieve 80% KTP removal throughout the treatment (Torán et al., 2017).

White-rot fungi of the *Pleurotus* spp. were also found to express lignin-cellulolytic enzymes, primarily laccases, throughout their life cycle, making them promising biodegraders of pharmaceutical pollutants. In a study by Cruz-Ornelas et al. (2019), *Pleurotus djamor* ECS-0123 demonstrated the ability to degrade diclofenac, naproxen, and KTP individually and in mixtures. These NSAIDs were added to the medium individually at 10 mg/L or in a pharmaceutical mixture at 7 mg/L each. The results showed that after 48 h of incubation, the fungus eliminated 87% of KTP. However, in the presence of naproxen, the degradation of both drugs decreased even after an extended exposure time of 72 h, with 85% degradation for naproxen and 83% for KTP. Notably, the degradation of KTP in a combination with both diclofenac and naproxen was considerably effective, with 68 and 83% degradation observed after 6 and 48 h, respectively. The study also indicated that laccase activity increased by 25% with the addition of KTP and by 300% with the addition of the NSAID mixture (Cruz-Ornelas et al., 2019).

*Pleurotus ostreatus*, isolated from a decaying wood fruiting body, exhibited moderate activity in the degradation of NSAIDs (Palli et al., 2017). In a fluidized bed bioreactor using hospital wastewater, the partial degradation of KTP (10 mg/L) reached 36% within 48 h. With continuous input of hospital wastewater, the removal of KTP increased to 60% within 2 days and 70% within 15 days. Two KTP decomposition products were identified using NMR spectroscopy (see Figure 2): 2-[3-(4-hydroxybenzoyl)phenyl] propanoic acid and 2-[(3-hydroxy(phenyl)methyl)phenyl]-propanoic acid formed through hydroxylation of the aromatic ring and reduction of the keto group, respectively.

Dalecka et al. (2020) reported that *Trametes versicolor* DSM 6401, *Trichoderma reesei* DSM 768, *Irpex lacteus* IBB 104, *Pleurotus ostreatus* DSM 1020 and *Fusarium solani* (an environmental isolate from a pharmaceutical WWTP located in Riga, Latvia) removed diclofenac and KTP from synthetic wastewater. *Trametes versicolor* DSM 6401 removed KTP (5 mg/L) by more than 80% within 21 days, while other strains and their mixtures did not exhibit any activity towards KTP (Dalecka et al., 2020). In a later study, *Trametes versicolor* DSM 6401 and *Aspergillus luchuensis* (an isolate from a municipal WWTP located in Stockholm, Sweden) were used to remove KTP from urban wastewater in a fluidized bed pelleted bioreactor (Dalecka et al., 2021). The study showed that both fungi removed KTP (50 mg/L), achieving an over 90% removal rate. Furthermore, *Trametes versicolor* DSM 6401 and *Aspergillus luchuensis* accomplished the pharmaceutical removal through biosorption mechanisms, which could provide important biotechnological advantages by avoiding byproduct synthesis and minimizing energy costs.

When considering members of Ascomycota as potential degraders of pharmaceutical pollutants, Ledezma-Villanueva et al. (2022) isolated a strain of *Cladosporium cladosporioides* from sewage sludge that exhibited the ability to degrade KTP. The strain successfully eliminated 90% of KTP (100 μM) for 21 days. In a subsequent study, the authors constructed a pilot-scale two-stage mesophilic anaerobic digestion system to remove pharmaceutical pollutants from sewage sludge. They selected an artificial microbial consortium composed of the fungi *Penicillium oxalicum* XD-3.1, *Penicillium raistrickii* and *Alternaria alternata*, *Cladosporium cladosporioides* and the bacterium *Micrococcus yunnanensis*. This consortium eliminated 100 μM KTP within 10 days, resulting in the production of two nontoxic metabolites: (2-[(3-hydroxy(phenyl)methyl)phenyl]-propanoic acid and (3-ethylphenyl)(phenyl)methadone (Figure 2) (Angeles-de Paz et al., 2023).

Unfortunately, working with fungi for the elimination of pharmaceutical pollutants from natural environments can be ineffective. In a study by Olicón-Hernández et al. (2021), *Penicillium oxalicum* XD-3.1 was employed in a fluidized batch bioreactor to treat real hospital wastewater containing KTP. The results were discouraging as only a minimal reduction in KTP concentration was achieved. It is worth noting that the wastewater also contained other NSAIDs such as diclofenac, ibuprofen, mefenamic acid, and naproxen, along with various antibiotics. The initial concentration of KTP in the wastewater was 9455 ± 289 ng/L, and during the 96 h of the bioreactor operation, the XD-3.1 strain showed modest removal of KTP (2.6%). Additionally, *Penicillium* *oxalicum* XD-3.1 inhibited local fungal populations, including opportunistic human pathogens such as *Mycosphaerella* and *Drechslera* (Olicón-Hernández et al., 2021).

Although the utilization of fungi as biodegraders of KTP shows promise, further extensive studies are needed to fully explore the potential and limitations of this approach. Identification of degradation metabolites and evaluation of their toxicity will enhance our understanding of the efficacy and safety of employing fungi in the biodegradation of KTP and other pharmaceutical pollutants.

### 4.2. Bacterial degradation of ketoprofen

Bacteria are known for their ability to metabolize a broad spectrum of recalcitrant compounds and are preferred over fungi in biotechnological processes. This preference is largely due to bacteria’s non-mycelial growth, which mitigates concerns related to biomass accumulation on internal bioreactor surfaces (Tyumina et al., 2020). Currently, several documented studies have explored the use of both individual bacterial strains and microbial consortia for the removal of KTP. The utilization of individual bacterial cultures not only enables the complete elimination of pollutants but also facilitates the identification of biodegradation products and metabolic pathways. Furthermore, it allows for a detailed examination of the mechanisms governing the interaction between bacterial cells and pharmaceutical pollutants along with the identification of relevant bacterial adaptation mechanisms (Ivshina et al., 2019, 2021a,b).

In a study by Palyzová et al. (2018), the efficiency of bacterial degradation of several analgesic substances including codeine, diclofenac, ibuprofen, and KTP was examined. The researchers discovered that a gram-negative strain named *Raoultella* sp. KDF8, which was isolated from composted waste, exhibited the capability to degrade KTP (1 g/L) by approximately 60 ± 1.76% within a 96-h period at 28°C (Palyzová et al., 2018).

Through a large-scale screening of stress-tolerant actinomycetes from the Regional Specialized Collection of Alkanotrophic Microorganisms [acronym IEGM, the large-scale research facilities (USU) #73559, the core facilities (CKP) #480868, the World Federation for Culture Collections (WFCC) #285; http://www.iegmcol.ru], we identified *Rhodococcus erythropolis* IEGM 746 as a strain capable of degrading approximately 40% of 100 mg/L KTP withinn 14 days (Bazhutin et al., 2022). Respirometric analysis confirmed the high catalytic activity of rhodococci in the presence of KTP, while atomic force microscopy revealed morphometric changes in cells induced by the NSAID.

The use of bacterial consortia can increase the number of involved catabolic pathways, thereby enhancing the efficiency of removal and biodegradation of pharmaceutical pollutants and even leading to their complete mineralization (Wojcieszyńska et al., 2014; Tripathi et al., 2021). Ismail et al. (2016) investigated the potential of artificial consortia for removing KTP as the sole carbon source. They found that a consortium of strains, including *Raoultella ornithinolytica* B6, *Pseudomonas aeruginosa* JPP, *Pseudomonas* sp. P16 and *Stenotrophomonas* sp. 5LF 19TDLC, which were isolated from wastewater, could degrade KTP (5 μM) at a rate of 33 μg/h, achieving the complete removal within 48 h (Ismail et al., 2016). The bioconversion process resulted in the formation of three by-products: (3-ethylphenyl)(phenyl)methanone, (3-hydroxyphenyl)(phenyl)methanone, and (3-hydroxyphenyl)(oxo)acetic acid. Their structural formulas are presented in Figure 2.

In recent years, there has been a growing interest in a system that combines microalgae and bacteria due to its versatility. This approach not only enables the purification of wastewater but also helps to reduce the cost of aeration. Moreover, the biomass generated from this system can have various applications, including animal feed production and utilization in the medical, pharmaceutical, and industrial sectors (Wang et al., 2013). Building upon their previous research, Ismail et al. (2017) further investigated the potential of a consortium comprising bacterial biomass supplemented with the green microalgae *Chlorella* sp. Iso4. This consortium decomposed KTP at a rate of 3.5 μg/h for 10 days after a lag phase of approximately 24 h. Subsequently, the authors used the same technology in a photobioreactor and combined four gram-negative bacterial strains with microalgae to remove a mixture of KTP (0.5 mM), aspirin/salicylic acid (0.5 mM), and paracetamol (0.4 mM). Throughout the degradation period (28 days), p-aminophenol, an intermediate product of paracetamol, was observed. It is important to note that p-aminophenol is known to be mutagenic and toxic. However, this observation did not impede the nearly complete biodegradation (97%) of the NSAID cocktail within 4 days of hydraulic retention (Ismail et al., 2017).

### 4.3. Degradation of ketoprofen by natural microbial communities

The conventional methods used to evaluate the bacterial degradation potential, which rely on cultivation-based approaches, underestimate the extensive diversity present in natural ecosystems and hinder the understanding of complex microbial assemblages. Recognizing the significant influence of microbial activity, abundance, diversity, and community structure on bioremediation strategies, researchers have begun to focus more on the ability of natural microbial communities, particularly those found in WWTPs, to degrade pharmaceuticals and the changes they undergo when exposed to these compounds (Siles and Margesin, 2018; Bôto et al., 2021).

One area of research has focused on the metabolism of KTP by ammonia-oxidizing bacteria. In a study conducted in Kitakyushu, Japan, biomass was collected from an aerobic tank in a WWTP and then used in a membrane bioreactor to break down a range of targeted pharmaceuticals and personal care products (45 compounds) in two stages. In the first stage, nitrifying bacteria oxidized ammonia to nitrate, facilitating the cometabolism of certain pollutants through non-specific enzymes, such as ammonia monooxygenases. In the second stage, after ammonia consumption, the targeted compounds, including KTP, were degraded alongside endogenous respiration (decay) in the absence of external organic matter or ammonia. The study reported a KTP biodegradation rate coefficient of 1.713 L/g VSS (volatile suspended solids) day (Park et al., 2017).

In a recent study on the removal of KTP, a sulfur-driven autotrophic denitrification bioreactor was employed. The biotransformation of KTP (100 μg/L) occurred over a period of 120 days and was initiated by the restoration of the ketone group through a series of oxidation/reduction and hydroxylation reactions. This process resulted in the formation of five intermediate products (see Figure 2): (3-(1-hydroxyethyl)phenyl)(phenyl)methanone, (3-(1-hydroxyethyl)phenyl)(3-hydroxyphenyl)methanone, (3-hydroxyphenyl)phenyl)methanone, 2-(3-(carboxy(hydroxy)methylphenyl)propanoic acid, and 2-(3-(carbonylcarbonyl)phenyl)-3-hydroxypropanoic acid. Furthermore, *Methyloversatilis*, *Methylotenera*, and *Hyphomicrobium* were identified as the dominant genera involved in the process of KTP biotransformation, with an efficiency of 55.4% ± 0.7%. At lower initial concentrations of KTP (25 and 50 μg/L), complete degradation was achieved within 5 days (Huang et al., 2023).

In another study conducted by Escuder-Gilabert et al. (2018), activated sludge collected from the aerated tanks of municipal WWTPs in Valencia, Spain, was utilized for the bioconversion of racemic ibuprofen and KTP at concentrations of 100 mg/L each. The bacterial degradation of KTP was completed after 25 days of incubation. Chromatographic analysis showed similar peak areas and biodegradation values for both enantiomers, indicating that the biodegradation of KTP by microorganisms in this case was apparently not enantioselective (Escuder-Gilabert et al., 2018).

Membrane bioreactors are a well-established technology in water management for wastewater treatment and reuse. These systems offer advantages such as the production of high-quality effluent, compact footprint, and minimal sludge generation (Gurung et al., 2019). Membrane bioreactors integrate a biological process with membrane filtration, where biomass biodegradation occurs within the bioreactor tank, and the separation of treated wastewater from microorganisms takes place within a membrane module (Al-Asheh et al., 2021). In the context of KTP removal, membrane bioreactors have been successfully applied, achieving almost complete removal (97.4%) of KTP at concentrations of about 200 ng/L from real wastewater (Gurung et al., 2019).

Few studies have been conducted to investigate the removal of KTP under anaerobic conditions. In an anaerobic membrane bioreactor, the elimination of hydrophilic compounds, including KTP, was found to be low (up to 40%). However, the addition of biochar enhanced the removal process (Lei et al., 2022). Another study documented a low degree of KTP removal (<20%) in the sulfate-reducing bacteria sludge system (Jia et al., 2019). The use of anaerobic granular sludge systems did not result in any KTP removal (Faria et al., 2022). In a sulfidogenic fluidized bed bioreactor used to treat acidic metal-containing water and hospital wastewater, the rate of KTP removal was only 41.9% (Makhathini et al., 2021). In a separate study involving a two-stage mesophilic anaerobic digestion process (acidogenic and methanogenic, connected in series), the removal of KTP from wastewater was 53% (Gallardo-Altamirano et al., 2021). Interestingly, the relative abundance of the class *Clostridia* (*Bacillota*) as well as the families *Propionibacteriaceae* (*Actinomycetota*) and *Cytophagaceae* (*Bacteroidota*), and the versatile methanogenic genus *Methanosarcina*, showed strong correlations with the presence of KTP in the system.

### 4.4. Enzyme-based degradation of ketoprofen

Enzyme bioremediation is a process of using naturally occurring enzymes to break down and degrade pollutants in the environment. Laccases (EC 1.10.3.2) are multi-copper oxidoreductases that catalyze the oxidation of numerous persistent organic pollutants and have proven to be effective in degrading various pharmaceuticals, including ibuprofen, naproxen, and diclofenac (Yang et al., 2017). The use of laccases in pharmaceutical biodegradation is an eco-friendly and cost-effective solution for reducing the environmental impact of pharmaceutical residues. Furthermore, the biodegradation process facilitated by laccases is selective and specific, providing a targeted approach for removing pharmaceuticals from the environment.

Recent studies have focused on the immobilization of laccases to enhance their efficiency in biodegradation. A study by Apriceno et al. (2019) investigated the potential use of chitosan-immobilized laccases from the white-rot fungus *Trametes versicolor* for the biodegradation of commonly encountered micropollutants such as KTP, diclofenac, and naproxen. The research findings revealed that the use of a biocatalyst facilitated the removal of 23% of KTP (100 mg/mL) within 7 days.

A similar study was conducted using a microfilter composed of hollow polysulfone fibers with tyrosinases and laccases from *Trametes versicolor* for the removal of 14 pharmaceuticals (antibiotics, NSAIDs, etc.) in a novel hybrid bioreactor (Ba et al., 2018). The findings demonstrated that this system effectively eliminated most pharmaceutical pollutants, irrespective of the dominant removal mechanism, whether enzymatic or sorption-based. Specifically, for KTP (10 μg/L), a remarkable 95% transformation was achieved within 24 h. Although the precise mechanisms underlying the removal of KTP were not fully elucidated, the authors proposed that the bioreactor’s membrane may contribute to heterogeneous catalysis by adsorbing the targeted pharmaceuticals and other compounds from wastewater.

Another study showed that the oxidative activity of laccase towards KTP and aspirin (25 mg/L) increased through adsorption immobilization of the enzyme on date stones (Al-sareji et al., 2023). In addition to achieving nearly complete removal of the pharmaceuticals within 4 h, laccase exhibited high activity over several cycles. This study highlights the significance of recycling, particularly the use of agricultural waste as adsorbents in biotechnology.

A novel approach involved the development of biocatalytic membranes by incorporating commercially available cellulose membranes with oxidoreductase enzymes, including laccase, tyrosinase, and horseradish peroxidase. These membranes were utilized for the removal of micropollutants from real wastewater samples (Zdarta and Zdarta, 2022). The findings from the study revealed that the laccase-membrane system exhibited the highest efficacy in reducing KTP. Moreover, the catalysts developed in this study demonstrated promising characteristics for storage and reuse, underscoring their practical applicability and potential for sustainable use in water treatment processes.

Nevertheless, not all studies have yielded positive results regarding the efficiency of immobilized enzymes in removing KTP. For instance, Jahangiri et al. (2018) conducted a study where they employed electron beam irradiation to cross-link laccase derived from *Phoma* sp. UHH 5-1-03 onto polyvinylidene fluoride membranes. Unfortunately, both batch and continuous treatments for the removal of KTP (10 μM) from wastewater resulted in only 15 and 2% removal, respectively.

The degradation of KTP by microorganisms, including fungi and bacteria, has been extensively studied. Fungi, particularly white-rot fungi such as *Trametes* spp., *Pleurotus* spp., *Phanerochaete* spp., and *Irpex* spp., have shown effective degradation of KTP, either individually or in combination with other pharmaceutical pollutants. Isolated enzymes found in fungi, such as laccases with their high oxidation activity, can also be efficiently utilized. As for bacteria, they have exhibited less biodegradation activity. However, the use of gram-negative bacterial consortia can significantly enhance the rate of KTP biodegradation. Additionally, promising approaches such as immobilization techniques, bacterial consortia, and combinations with microalgae have shown potential in improving the removal efficiency of KTP.

Therefore, it is important to note that while there are effective methods for removing KTP from wastewater using whole-cell and enzyme biocatalysts, the potential formation of toxic byproducts should not be overlooked. This highlights the importance of not only finding efficient ways to remove micropollutants but also considering the potential environmental impact of the removal process itself. Ultimately, the pharmaceutical industry needs to implement more responsible disposal strategies to minimize the overall impact of these compounds on the environment.

### 4.5. Algal adsorption of ketoprofen

In addition to biodegradation, sorption and adsorption technologies offer effective means of removing pharmaceutical pollutants from water. Sorption and adsorption technologies involve the physical binding of pharmaceutical contaminants to solid surfaces or materials. Common sorbent materials include algal biomass, activated carbon and other porous materials with high surface areas.

The application of adsorption processes, including biosorption, has shown a high potential for the eliminations of pharmaceuticals (Georgin et al., 2022). Although the use of algae for the removal of NSAIDs is a relatively new area of research, several studies have reported their effectiveness in addressing pharmaceutical contamination. In particular, macroalgae are mainly used to treat waste generated during pharmaceutical manufacturing since they serve as a carbon substrate for the formation of a porous and robust material, acting as an adsorbent (Boakye et al., 2019). For example, Ouasfi et al. (2019) investigated the biosorption of KTP from an aqueous solution using brown seaweed *Laminaria digitata*, which was collected in El Jadida on the West Atlantic coast of Morocco. The maximum adsorption capacity for KTP (150 mg/L) at 25°C and pH 3.4 was 443.45 mg/g, and its biosorption efficiency within 1 hour was 86.12% (Ouasfi et al., 2019).

Microalgae have also been explored for their potential in eliminating pharmaceutical pollutants. Hifney et al. (2021) conducted a study that specifically examined the use of *Chlorella* sp. cells for the adsorption of diclofenac (2.5 mg/L) and KTP (5 mg/L) from aqueous solutions. The *Chlorella* sp. culture used in the study was isolated from a polluted site in Assiut Governorate, Egypt. It is worth noting that the *Chlorella* genus is recognized for its high resistance to various stressful conditions. The study demonstrated that the adsorption of KTP onto the cells was initially slow, but it significantly increased over time as the contact time between the pharmaceutical compound and *Chlorella* sp. cells increased. Furthermore, over 75% of diclofenac and 60% of KTP were removed within 90 and 60 min, respectively. However, a gradual decrease in adsorption efficiency was observed thereafter due to the desorption phenomenon. This observation emphasized the significance of the contact time as a critical parameter in achieving optimal removal of xenobiotics and preventing the process of desorption over extended periods (Godos et al., 2009; Hifney et al., 2021).

### 4.6. Phytoremediation of ketoprofen

Phytoremediation, also known as “plant-based remediation,” utilizes the natural abilities of plants to clean up the environment. Plants are capable of extracting, accumulating, and detoxifying the contaminants present in soil, air, and water through various physical, chemical, and biological processes.

Plant-assisted bioremediation of water contaminants is primarily utilized in constructed wetlands, which are water treatment systems that employ microbial communities, wetland plants, and natural soil processes to enhance water quality. In Tuscany, Italy, constructed wetlands planted with herbaceous species *Phragmites australis* (common reed) and trees *Salix matsudana* (willow) were used for removing nonylphenols and pharmaceuticals (atenolol, diclofenac, KTP, triclosan) from wastewater (Francini et al., 2018). The presence of both plants resulted in an over 80% removal efficiency of KTP. However, as noted by the authors, the removal of KTP from the water was due to its accumulation in the plants and sorption on gravel. In addition, the research demonstrated that KTP was absorbed by willow (125 ng/g) at greater levels compared to common reed (75 ng/g). Ren et al. (2023) investigated the removal of KTP in constructed wetlands under various saturation conditions, and the efficiency of KTP removal was 50–99.7%. Sánchez et al. (2022) constructed a pilot-scale system comprising a hybrid digester as the first step, a subsurface vertical flow constructed wetland as the second step with effluent recirculation, and a photodegradation unit as the third step/post-treatment. Using that system, KTP and other NSAIDs were completely removed (>99.5 %). The high effectiveness of KTP elimination from real wastewater using aerated constructed wetlands was also confirmed by Ávila et al. (2021).

Indeed, while adsorption and phytoaccumulation are effective in removing pharmaceutical pollutants, they may not eliminate all contaminants entirely. Other substances present in water can compete with pharmaceuticals for adsorption sites, potentially reducing the elimination efficiency. Adsorption itself is a non-selective process, meaning it does not specifically target particular pollutants but instead relies on the affinity between the adsorbent material and a wide range of molecules.

Furthermore, the bioaccumulation of xenobiotics in macrophytes can pose ecological risks, particularly if these plants are consumed by herbivores or if they decompose and release the accumulated compounds back into the environment. Additionally, the persistence of these compounds within plant tissues may limit the overall effectiveness of phytoremediation because bioaccumulated xenobiotics can remain in the system even after the macrophytes are harvested or decay.

To address these limitations, it may be useful to combine adsorption and bioaccumulation with other treatment techniques, such as biological degradation or advanced oxidation processes, to enhance the overall removal efficiency. By employing a multi-step approach, it becomes possible to target a broader spectrum of pollutants and achieve more comprehensive remediation of contaminated environments.

## 5. Conclusion

In light of the growing emphasis on environmental sustainability, monitoring of pharmaceutical pollutants, including those from hospital and municipal wastewater, has become crucial. Efforts are being made to establish observatories and promote a circular economy where valuable substances can be recovered from WWTPs instead of being discarded.

This review has examined the environmental pollution caused by KTP, including its occurrence, fate, removal and biodegradation, and associated risks. While abundant studies have investigated the detection of KTP in aquatic environments, our understanding of its presence in soil, terrestrial flora and fauna is limited. Consequently, comprehensively assessing the true extent of its toxic effects and the implications of its accumulation in ecosystems poses a challenge. Moreover, additional research is needed to explore the potential toxicity of KTP degradation products and their interactions with other xenobiotics.

Although KTP is generally considered persistent in the environment, recent studies have shown its potential for biodegradation, suggesting that natural attenuation processes may contribute to its reduction. Fungi have been extensively investigated for KTP biodegradation, but research on bacteria, plants, algae, and specific enzymes is emerging as potential avenues for KTP removal. However, the efficacy of KTP removal depends on various parameters, including its physico-chemical characteristics. The complex chemical composition of KTP contributes to its poor sorption and high desorption abilities, and the presence of certain functional groups can reduce its susceptibility to oxidative catabolism.

Despite the considerable progress, there are still gaps in our knowledge regarding the degradation of KTP. It is crucial to identify and characterize the decomposition products of KTP and to further investigate the role of microbial communities in the degradation process. These efforts will contribute to a better understanding of the environmental impact of KTP and enhance the efficiency of its removal.

## Author contributions

II coordinated the work, secured research funding, reviewed, and edited the manuscript. ET drafted the manuscript. MS, MP, and ST contributed to data collection, analysis, and manuscript preparation. All authors contributed to the article and approved the submitted version.

## Funding

This work was supported by the Russian Science Foundation grant (no. 21-14-00132).

## Conflict of interest

The authors declare that the research was conducted in the absence of any commercial or financial relationships that could be construed as a potential conflict of interest.

## Publisher’s note

All claims expressed in this article are solely those of the authors and do not necessarily represent those of their affiliated organizations, or those of the publisher, the editors and the reviewers. Any product that may be evaluated in this article, or claim that may be made by its manufacturer, is not guaranteed or endorsed by the publisher.
