# Supplementary material for: Ketoprofen as an emerging contaminant: occurrence, ecotoxicity and (bio)removal
Source: Front Microbiol. 2023 Aug 7;14:1200108. doi: 10.3389/fmicb.2023.1200108 (PMC10441242; doi:10.3389/fmicb.2023.1200108)
Supplement: Supplementary file 1 [file Image_1.PDF]

Supplementary Material

Ketoprofen as an Emerging Contaminant: Occurrence, Ecotoxicity and (Bio)removal

Elena Tyumina*, Maria Subbotina, Maxim Polygalov, Semyon Tyan, Irina Ivshina

*** Correspondence:** Elena Tyumina: tyumina@psu.ru

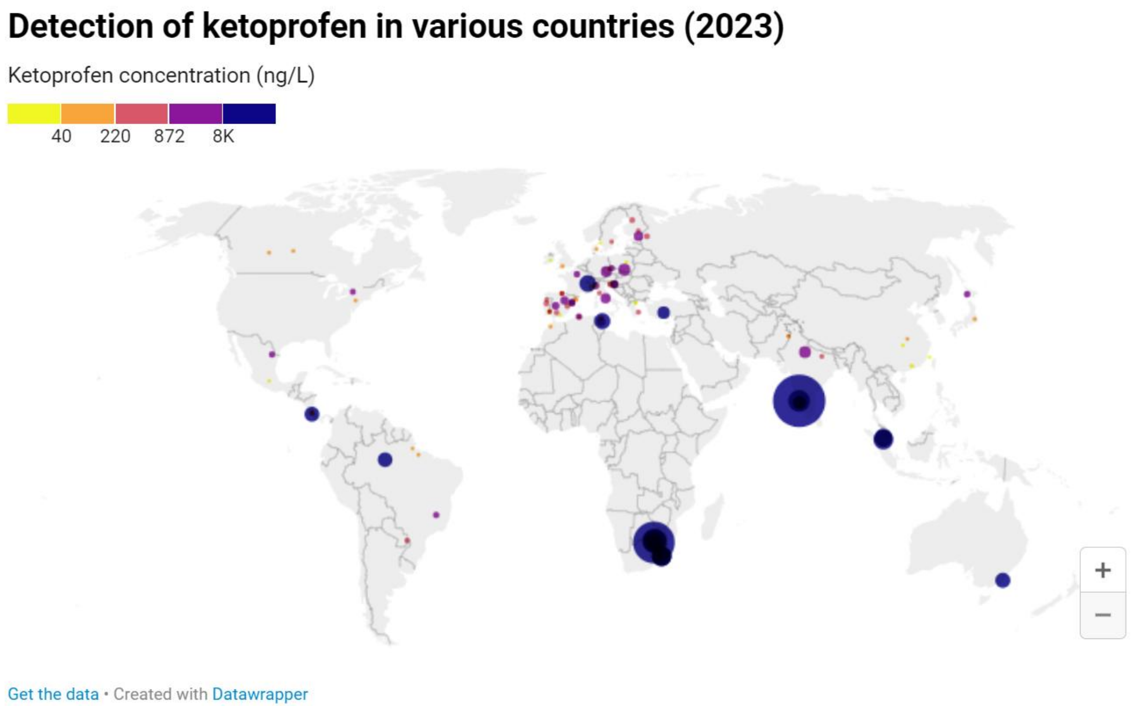

Supplementary Figure 1. Detection of ketoprofen based on data from Table 1. Dots on the map represent specific sampling locations (https://datawrapper.dwcdn.net/MgAbF/8/).
